# Trajectory of cardiac troponin T following moderate-to-severe COVID-19 and the association with cardiac abnormalities

**DOI:** 10.1186/s12872-024-03854-7

**Published:** 2024-04-13

**Authors:** Tarjei Øvrebotten, Albulena Mecinaj, Knut Stavem, Waleed Ghanima, Eivind Brønstad, Michael T Durheim, Tøri V. Lerum, Tony Josefsen, Jostein Grimsmo, Siri L. Heck, Torbjørn Omland, Charlotte B. Ingul, Gunnar Einvik, Peder L. Myhre

**Affiliations:** 1 Department of Cardiology, Division of Medicine, Akershus University Hospital, Lørenskog, Norway; 2 K.G. Jebsen Center for Cardiac Biomarkers, Institute for Clinical Medicine, University of Oslo, Oslo, Norway; 3 Health Services Research Unit, Akershus University Hospital, Lørenskog, Norway; 4 Department of Pulmonary Medicine, Akershus University Hospital, Lørenskog, Norway; 5 Institute of Clinical Medicine, Faculty of Medicine, University of Oslo, Oslo, Norway; 6 Department of Hemato-oncology, Østfold Hospital Kalnes, Østfold, Norway; 7 Department of Circulation and Medical Imaging, Norwegian University of Science and Technology, Trondheim, Norway; 8 Thoracic Department, St. Olavs Hospital, Trondheim, Norway; 9 Department of Respiratory Medicine, Oslo University Hospital Rikshospitalet, Oslo, Norway; 10 Department of Pulmonary Medicine, Oslo University Hospital Ullevål, Oslo, Norway; 11 Department of Cardiology, Østfold Hospital Kalnes, Østfold, Norway; 12 Department of cardiac and pulmonary rehabilitation, Cathinka Guldberg’s Hospital, Lovisenberg Rehabilitation, Jessheim, Norway; 13 Department of Diagnostic Imaging, Akershus University Hospital, Lørenskog, Norway

**Keywords:** COVID-19, Troponin, Echocardiography, Cardiac function, Biomarker

## Abstract

**Background:**

COVID-19 has been associated with cardiac troponin T (cTnT) elevations and changes in cardiac structure and function, but the link between cardiac dysfunction and high-sensitive cardiac troponin T (hs-cTnT) in the acute and convalescent phase is unclear.

**Objective:**

To assess whether hs-cTnT concentrations are associated with cardiac dysfunction and structural abnormalities after hospitalization for COVID-19, and to evaluate the performance of hs-cTnT to rule out cardiac pathology.

**Methods:**

Patients hospitalized with COVID-19 had hs-cTnT measured during the index hospitalization and after 3-and 12 months, when they also underwent an echocardiographic study. A subset also underwent cardiovascular magnetic resonance imaging (CMR) after 6 months. Cardiac abnormalities were defined as left ventricular hypertrophy or dysfunction, right ventricular dysfunction, or CMR late gadolinium.

**Results:**

We included 189 patients with hs-cTnT concentrations measured during hospitalization for COVID-19, and after 3-and 12 months: Geometric mean (95%CI) 13 (11–15) ng/L, 7 (6–8) ng/L and 7 (6–8) ng/L, respectively. Cardiac abnormalities after 3 months were present in 45 (30%) and 3 (8%) of patients with hs-cTnT ≥ and < 5 ng/L at 3 months, respectively (negative predictive value 92.3% [95%CI 88.5–96.1%]). The performance was similar in patients with and without dyspnea. Hs-cTnT decreased from hospitalization to 3 months (more pronounced in intensive care unit-treated patients) and remained unchanged from 3 to 12 months, regardless of the presence of cardiac abnormalities.

**Conclusion:**

Higher hs-cTnT concentrations in the convalescent phase of COVID-19 are associated with the presence of cardiac pathology and low concentrations (< 5 ng/L) may support in ruling out cardiac pathology following the infection.

**Supplementary Information:**

The online version contains supplementary material available at 10.1186/s12872-024-03854-7.

## Introduction

Cardiac troponin T (cTnT) is released into the circulation following myocardial injury and elevated concentrations are associated with an increased risk of cardiovascular events in the general population and among patients with cardiovascular disease (CVD ) [1, 2]. Cardiac remodeling, particularly left ventricular (LV) hypertrophy, myocardial scar, and diastolic dysfunction are associated with higher concentrations of cTnT [2–4]. Patients hospitalized with COVID-19 frequently have cTnT concentrations above the upper reference limit in the acute phase of the infection [5]. Elevated cardiac troponin concentrations during COVID-19 are associated with poor outcomes within one year in retrospective studies [6–8], while prospective studies with unselected patients suggest that the prognostic information is modest and not independent of established risk factors [9].

The prevalence and severity of cardiac pathology after COVID-19 vary depending on the setting and patient selection (in-hospital, outpatient, athletes) and sensitivity of the imaging modality [10–15]. Many COVID-19 patients report symptoms such as persistent dyspnea and fatigue beyond 4 weeks (i.e. Post-Acute COVID Syndrome [PACS]). The degree to which cardiac dysfunction may contribute to these symptoms remains to be determined.

Very low levels of high-sensitive cTnT (hs-cTnT) are associated with a low likelihood of cardiac remodeling in the general population and patients with CVD [2, 16]. Whether hs-cTnT concentrations in the acute and convalescent phase of COVID-19 are associated with subsequent abnormal cardiac function is unclear. We hypothesized that concentrations of hs-cTnT were associated with cardiac pathology after hospitalization for COVID-19, and that very low hs-cTnT concentrations have a high negative predictive value (NPV) for cardiac disease. We aimed to assess this by analyzing a cohort of unselected patients hospitalized with COVID-19 during the first wave of the pandemic in Norway. Patients who required treatment at the ICU and patients with persistent dyspnea were of particular interest, and analyzed separately. We also aimed to establish the trajectory of hs-cTnT concentrations from hospitalization to 3 and 12 months after hospital discharge, and its association with cardiac pathology.

## Methods

### Study design and population

Unselected patients hospitalized with COVID-19 between February 2020 and June 2020 were included in Patient-Related Outcomes and Lung Function after Hospitalization for COVID-19 (PROLUN), a prospective multi-center cohort study at six major hospitals in Norway [17–19]. Inclusion criteria were age > 18 years, admission lasting for ≥ 8 h with a diagnosis of COVID-19, or a positive SARS-CoV-2 PCR test combined with viral pneumonia. All included patients were invited to a follow-up visit approximately 3 months (median 102 days) and 12 months (median 387 days) after their index hospitalization. The follow-up visits were performed by dedicated study personnel at each study center from June 1 to August 28, 2020 (3 months visit) and from February 26 to June 29, 2021 (12 months visit).

A subgroup of PROLUN participants was concurrently included in another prospective, observational study (COVID-MECH; NCT04314232) where they were invited to a cardiac magnetic resonance (CMR) examination approximately 6 months (median 175 days) after the COVID-19 hospitalization (between June 24 and November 18, 2020) [20].

### Echocardiography

Echocardiographic imaging was performed according to standard guideline recommendations by five experienced operators following a predefined protocol [17, 18]. All images were recorded using Vivid E95 GE. The images were later analyzed by a single investigator to reduce inter-reader variability. Left ventricular (LV) global longitudinal strain and right ventricular (RV) free wall strain were measured with software from GE imaging systems, and measurements are presented in absolute values.

LV hypertrophy was defined as left ventricular mass index (LVMi) > 115 g/m^2^ for men and > 95 g/m^2^ for women [21]. Systolic dysfunction was defined as LV global longitudinal strain < 18% *and* LV ejection fraction < 50% [22]. Diastolic dysfunction was defined as ≥ 2 of 3: Indexed left atrial (LA) volume ≥ 34 ml/m^2^, lateral E’<10 cm/s or septal E’ <7 cm/s, and E/e’ >14. Impaired RV function was defined as tricuspid annular plane systolic excursion (TAPSE) < 1.8 cm or RV free wall strain < 20% [21].

### Cardiovascular magnetic resonance imaging (CMR)

All patients in the COVID-MECH study were invited to the CMR substudy and 58 (45%) completed the examination, as previously described [20]. All CMR examinations were performed at Akershus University Hospital, using a 1.5 Tesla MRI scanner (Achieva; Philips Medical Systems, Best, The Netherlands) [20]. Myocardial scar was defined as the presence of late gadolinium enhancement (LGE) on CMR. LGE was obtained using phase-sensitive inversion recovery LGE imaging after injections of gadoterate meglumine (Clariscan® Gé, GE Healthcare). Minor myocardial scars were defined as the presence of scars without a concomitant reduction in LV ejection fraction (< 50%). Images were captured using 10 mm short-axis slices all through the ventricles, in addition to three long-axis views.

### Blood sampling and troponin measurements

Blood samples were collected and analyzed at several time points during the index hospitalization, and peak hs-cTnT was defined as the highest concentration recorded during the hospital stay. hs-cTnT was also measured in blood samples drawn at the visits 3 and 12 months after discharge. Blood samples were centrifuged at 4 °C, and the plasma was frozen and stored at each study site. At all locations, the analysis of cTnT was performed on a Cobas e801 platform with the high-sensitivity assay (cTnT hs STAT, Roche Diagnostics, Penzberg, Germany), except Trondheim University Hospital where it was analyzed on Cobas e602. This assay has an analytical range from 3 to 10 000 ng/L, a limit of detection (LoD) of 3 ng/L, and the upper reference limit (99th percentile in the general population) is 14 ng/L on both platforms [23]. Following the recommendations of the International Federation of Clinical Chemistry and Laboratory Medicine (IFCC), hs-cTnT values are reported in whole numbers.

We used a hs-cTnT threshold of 5 ng/L to stratify patients. This was adopted from the recommended cut-off used to rule myocardial infarction in suspected acute coronary syndrome, as recommended in the current European Society of Cardiology Guidelines [24].

### Assessment of dyspnea

Dyspnea was classified by the modified Medical Research Council (mMRC) dyspnea scale at the 3- and 12-month visits. The mMRC is a self-rating tool to measure the degree of disability that breathlessness poses on day-to-day activities on a scale from 0 (no dyspnea) to 4 (maximum dyspnea) [25]. We performed separate analyses with dyspnea defined as mMRC ≥ 1 (i.e. ‘dyspnea when hurrying or walking up a slight hill’) and mMRC ≥ 2 (‘Walks slower than people of the same age because of dyspnea or has to stop for breath when walking at own pace’).

### Sub-group analysis

In an attempt to study troponin in relation to disease severity, we stratified the patients based on whether they had been treated in the intensive care unit (ICU; with or without respiratory support) or admitted to the medical ward. Admission to the ICU was determined based on clinical assessment in the emergency department. ICU stay was registered retrospectively after inclusion in the study through review of the medical records.

We defined the severity of the COVID-19 infection based on whether the patient received treatment in the ICU (severe infection) or not (moderate infection).

### Statistical analysis

Continuous variables are presented as means ± standard deviation (SD). Since hs-cTnT is not normally distributed we used geometric mean (95% CI) to describe the distribution of concentrations. All other variables were investigated with histograms and found to be normally distributed, except creatinine which is presented as median (25th -75th percentile). Categorical variables are presented as absolute numbers and percentages. Groups were compared using the t-test, chi-square test, or Fisher’s exact test, as appropriate. Delta values for change in variables from 3 to 12 months were generated by subtraction of the first value from the last value. Patients were categorized according to hs-cTnT levels above or below 5 ng/L and compared using logistic regression in univariable and multivariable models including age, sex, and a history of cardiovascular disease. Log-transformed hs-cTnT concentrations were also analyzed as a continuous variable in multivariable linear regression models with effect estimates and 95% CI. Patients with hs-cTnT below the LoD (< 3 ng/L) were assigned the value of 1.5 ng/L for analytical purposes. Changes in hs-cTnT from hospitalization to 3 months and 12 months were analyzed by the Wilcoxon signed-rank test for paired samples. Comparisons between groups were determined using the Mann-Whitney U test.

We calculated the receiver operating characteristic area under the curve (ROC AUC) for hs-cTnT, and sensitivity, specificity, negative predictive (NPV), positive predictive value (PPV) and Likelihood ratio (LR) for the performance of hs-cTnT in detecting cardiac pathology when using a cut-off of 5 ng/L. We performed this analysis separately for participants with and without persistent dyspnea 3 months after COVID-19, defined as mMRC ≥ 1 and ≥ 2 in separate models. Statistics were performed using Stata version 16.1. P-values are 2-sided, and values less than 0.05 are considered significant.

## Results

### Patient characteristics

Of 204 patients in the PROLUN echocardiography study, 189 (93%) patients had hs-cTnT measured at the 3-month follow-up visit, and 58 (28%) patients who were also enrolled in the COVID MECH study had available CMR examination at the 6-month visit (Suppl. Figure 1). The mean age was 58.9 ± 13.7 years, 58% were male, 82% were Caucasian and 10% had pre-existing CVD (Table 1). At 3 months, 6 (3%) patients had LV hypertrophy, 8 (4%) had LV systolic dysfunction, 27 (16%) had diastolic dysfunction, and 7 (4%) had RV dysfunction. Of the 58 patients who underwent CMR, 12 (21%) had myocardial scars. One patient had a combined non-ischemic and ischemic (subendocardial) scar, while the rest had non-ischemic scars (myocardial and/or epicardial). Nine participants had scars that were classified as minor and three had scars that were classified as major. In total, 41 (22%) patients had one or more abnormal findings on echocardiography and this increased to 48 (25%) when including myocardial scar from the subset with CMR.

**Table 1:** Baseline characteristics of patients stratified by a high sensitivity cardiac troponin T concentration (hs-cTnT) of 5 3 months after hospitalization for COVID-19

	All *n* = 189	cTnT < 5 ng/L *n* = 39	cTnT ≥ 5 ng/L *n* = 150	P-value
Age at discharge, years	58.9 ± 13.7	48.0 ± 10.5	61.7 ± 13.1	<0.001
Male sex	110 (58%)	16 (41%)	94 (63%)	0.015
Caucasian ethnicity	154 (82%)	27 (69%)	127 (85%)	0.027
Body mass index, kg/m^2^	28.3 ± 4.6	29.3 ± 4.8	28.0 ± 4.6	0.13
Obesity (BMI > 30)	60 (32%)	15 (39%)	45 (30%)	0.31
Cardiovascular disease	19 (10%)	0	19 (13%)	0.002
Hypertension	63 (34%)	5 (13%)	58 (40%)	0.002
Diabetes	17 (9%)	3 (8%)	14 (9%)	0.51
Chronic obstructive pulmonary disease	6 (3%)	0	6 (4%)	0.35
Chronic kidney disease	3 (2%)	0	3 (2%)	0.50
Current or previous smoking	89 (47%)	13 (33%)	76 (51%)	0.05
**Hospital admission**				
Length of stay, days	6 [3,11]	6 [4,10]	6 [3,12]	0.92
Intensive care treatment	36 (19%)	8 (21%)	28 (19%)	0.79
Creatinine at admission, umol/L	75 [63, 92]	62 [54, 73]	79 [68, 94 ]	< 0.001

### Clinical predictors of higher levels of hs-cTnT 3 and 12 months after COVID-19

Three months after COVID-19, the geometric mean (95% CI) of hs-cTnT was 7 (6–8) ng/L. hs-cTnT concentrations ≥ 5 ng/L was present in 150 (79%) of participants, and these were older, more often men, and had higher serum creatinine concentration and a higher prevalence of CVD and hypertension, compared to those with hs-cTnT < 5 ng/L (Table 1). In multivariable models with log-transformed hs-cTnT at 3 months as the continuous outcome variable, age (*P* < 0.001) and male sex (*P* < 0.001) were the only independent predictors of higher concentrations (Suppl. Table 1). Age and sex were also the only significant predictors of hs-cTnT in multivariable models at 12 months.

### Association between hs-cTnT and cardiac structure and function 3 and 12 months after COVID-19

Log-transformed concentrations of hs-cTnT measured 3 and 12 months after COVID-19 were associated with the presence of cardiac abnormalities (P < 0.001 for both), and this association persisted after adjusting for age and sex (unstandardized beta (B) 0.34 (95% CI 0.13–0.55), at 3 months and B 0.30 (95% CI 0.87 − 0.52) at 12 months). Patients with concentrations of hs-cTnT ≥ 5 ng/L had worse cardiac structure and function, including greater LV mass and RV dimension and higher E/e’ (Table 2). After adjusting for age and sex, these associations persisted for LV mass (*P* = 0.02), but not for RV dimension and E/e’. hs-cTnT concentration at 3 months was numerically, but not significantly, higher in patients with myocardial scar (*n* = 12) than patients without myocardial scar: geometric mean (95% CI) 12 (8–20) ng/L versus 7 (6–8) ng/L, *P* = 0.07 (Table 2). The results were consistent in models excluding participants with pre-existing CVD (Suppl. Table 2). Hs-cTnT concentrations at 3 months were not associated with future changes in cardiac structure and function from 3 to 12 months (Suppl. Table 3), with consistent findings after excluding patients with pre-existing CVD (Suppl. Table 4).

**Table 2:** Measurements of cardiac structure and function in patients with high sensitivity cardiac troponin T (hs-cTnT) < 5 ng/L and ≥ 5 ng/L at 3-month follow-up

	hs-cTnT < 5 *n* = 39	hs-cTnT ≥ 5 ng/L *n* = 150	P-value	Adjusted P-value (Age + Sex + CVD*)
Left ventricular mass index (g/m^2^)	60 ± 13	73 ± 18	0.001	0.03
Left ventricular end-diastolic volume index (ml/m^2^)	50 ± 9	53 ± 14	0.21	0.26
Left ventricular ejection fraction (%)	58 ± 4	58 ± 6	0.39	0.69
Left ventricular global longitudinal strain (%)	19.9 ± 1.6	19.1 ± 2.4	0.10	0.21
Left atrial volume index (ml/m^2^)	24 ± 7	27 ± 9	0.06	0.59
E / e`	8.0 ± 0.5	8.4 ± 0.2	0.01	0.53
Right ventricular basal diameter (cm)	3.5 ± 0.6	3.8 ± 0.5	0.02	0.19
Tricuspid annular plane systolic excursion (cm)	2.4 ± 0.3	2.4 ± 0.3	0.71	0.76
Right ventricular free wall strain (%)	26.4 ± 2.8	25.8 ± 4.2	0.46	0.57
Estimated systolic pulmonary arterial pressure (mmHg)	25 ± 6	23 ± 9	0.34	0.036
Myocardial scar (Late gadolinium enhancement, *n* = 58)	1 (6%)	11 (27%)	0.07	0.58
Myocardial scar volume (%)	1.74	2.70 ± 1.75		

Reported as mean ± SD or number (%). *P*-values are calculated from logistic regression

*Cardiovascular disease

### hs-cTnT for ruling out cardiac abnormalities after COVID-19

Patients with hs-cTnT ≥ 5 ng/L 3 months after COVID-19 had higher odds of having ≥ 1 abnormal finding on echocardiography or CMR than those with hs-cTnT < 5 ng/L: OR 5.14 [95% CI 1.5–17.5], *P* = 0.009, but this did not persist after adjusting for sex and age (OR 2.0 [95% CI 0.53–7.55), *P* = 0.31) (Table 3). C-statistics for identifying cardiac pathology was 0.79 (95% CI 0.72–0.87). hs-cTnT concentrations < 5 ng/L yielded a NPV of 92.3% (95% CI 88.5–96.1%) and a negative LR of 76% (Suppl. Table 5). The results were consistent when excluding patients with established CVD (Suppl. Table 6).

**Table 3:** Number (%) of patients with cardiac abnormalities after COVID-19 assessed by echocardiography and cardiac magnetic resonance in patients with high-sensitivity cardiac troponin T (hs-cTnT) < 5 ng/L and ≥ 5 ng/L. Comparisons between the groups are performed with logistic regression

*Echocardiography (n = 189)*	hs-cTnT < 5 *n* = 39	hs-cTnT ≥ 5 *n* = 150
Left ventricular hypertrophy	0 (0%)	6 (4%)
Diastolic dysfunction	2 (6%)	25 (18%)
Systolic dysfunction	0 (0%)	8 (5%)
Right ventricular dysfunction	0 (0%)	7 (6%)
Abnormal cardiac findings with echocardiography	**2 (5%)**	**39 (26%)**
Unadjusted	OR 6.5 (95% CI 1.5–28.2) *P* = 0.013	
Adjusted for age, sex and cardiovascular disease	OR 2.6 (95% CI 0.5–12.6) *P* = 0.16	
***Cardiac magnetic resonance*** ***(n = 58)***	***n*** ** = 17**	***n*** ** = 41**
Myocardial scar (LGE)	1 (6%)	11 (27%)
Abnormal cardiac findings with echocardiography and cardiac magnetic resonance	**3 (8%)**	**45 (30%)**
Unadjusted	OR 5.1 (95% CI 1.5–17.6) *P* = 0.009	
Adjusted for age, sex and cardiovascular disease	OR 2.0 (95% CI 0.5–7.6) *P* = 0.22	

Among patients with persistent dyspnea (defined as mMMRC ≥ 1) 3 months after COVID-19 (*n* = 84, 44%), 20 (24%) had one or more cardiac abnormalities. The geometric mean (95% CI) concentration of hs-cTnT among patients with dyspnea was 6 (5–8) ng/L, which was not significantly different from those without dyspnea 6 (5–7) ng/L, *P* = 0.61. Among patients with dyspnea, hs-cTnT concentrations ≥ 5 ng/L were associated with an 8-fold higher risk of cardiac abnormality, compared to patients with hs-cTnT < 5 ng/L: OR 8.02 [95% CI 1.001–64.3], *P* = 0.05, but this did not persist in adjusted models (*P* = 0.28). The C-statistics for hs-cTnT for discriminating between those with and without cardiac abnormality in dyspneic patients was 0.80 and levels < 5 ng/L had NPV 96% (92–100%) with an LR- of 86%. When using a stricter definition of dyspnea (mMMRC ≥ 2) the C-statistics were 0.75, NPV 83% (75–92%), and LR- 80% (70-90.5%) (Suppl. Table 7). The results were consistent when excluding patients with CVD.

### Peak troponin during hospitalization for COVID-19 in association with cardiac structure and function in the convalescent phase

Peak hs-cTnT concentrations during hospitalization were available in 157 (83%) patients and the geometric mean (95% % CI) was 13 (11–15) ng/L for the entire population. Peak hs-cTnT was higher in patients treated in the intensive care unit (ICU) compared to those treated in the medical ward: 22 (14–34) ng/L versus 11 (9–13) ng/L, *P* < 0.001 (*P* = 0.004 after adjusting for age and sex). During the hospitalization for COVID-19, 8 patients died, and these patients had higher hs-cTnT levels than survivors attending the 3-month visit: geometric mean (95% CI) of 38 (23–65) vs. 13 (12–15), respectively (*P* = 0.002). Peak hs-cTnT concentrations during hospitalization predicted the presence of abnormal cardiac findings 3 months after COVID-19 among survivors (geometric mean [95% CI]): 17 (13–23) ng/L and 11 (9–14) ng/L, *p* = 0.034, for patients with and without cardiac abnormalities respectively). However, after adjusting for age and sex, this association was attenuated (*P* = 0.82). Similar to the cross-sectional associations 3 months after COVID-19, unadjusted analyses showed that higher peak hs-cTnT levels during hospitalization correlated with greater LV mass, greater LA volume, and higher E/e` after 3 months. Peak hs-cTnT levels during hospitalization were similar in patients with and without persisting dyspnea after 3 months (geometric mean [95% CI]: 14 (11–17) ng/L vs. 12 (9–17) ng/L, respectively, *P* = 0.52)

### Trajectories of hs-cTnT during COVID-19 and after 3 and 12 months

**Fig. 1:**
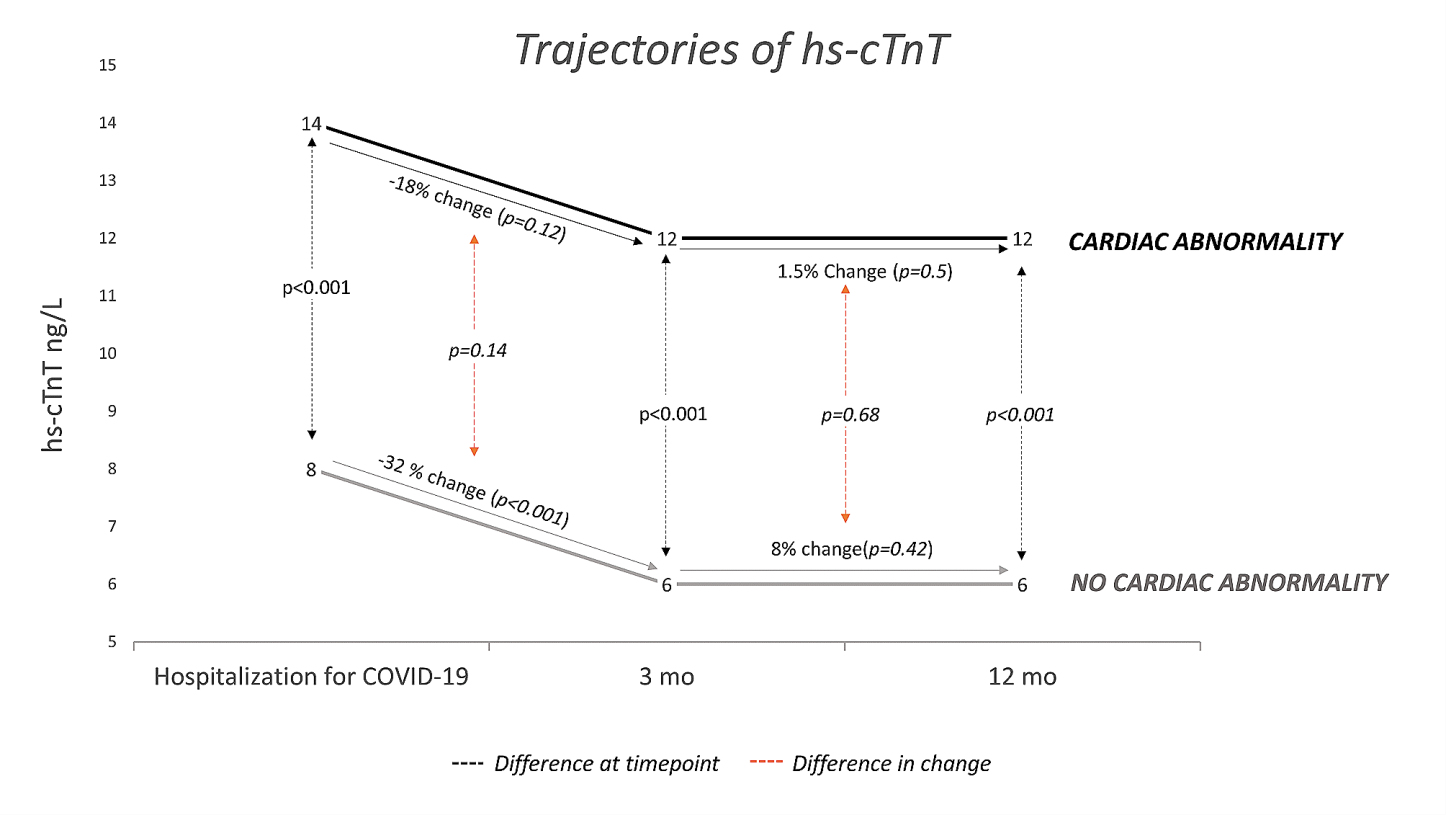
Trajectory of high sensitivity cardiac troponin T (hs-cTnT) from peak concentration during hospitalization for COVID-19 to 3 and 12 months after discharge. Stratified by the presence of cardiac abnormality after 3 months and presented as geometric mean. Patients with hs-cTnT available at all time points (*n* = 125). Hs-cTnT concentrations with the corresponding 95% confidence interval reported in Suppl.Table 8

**Fig. 2:**
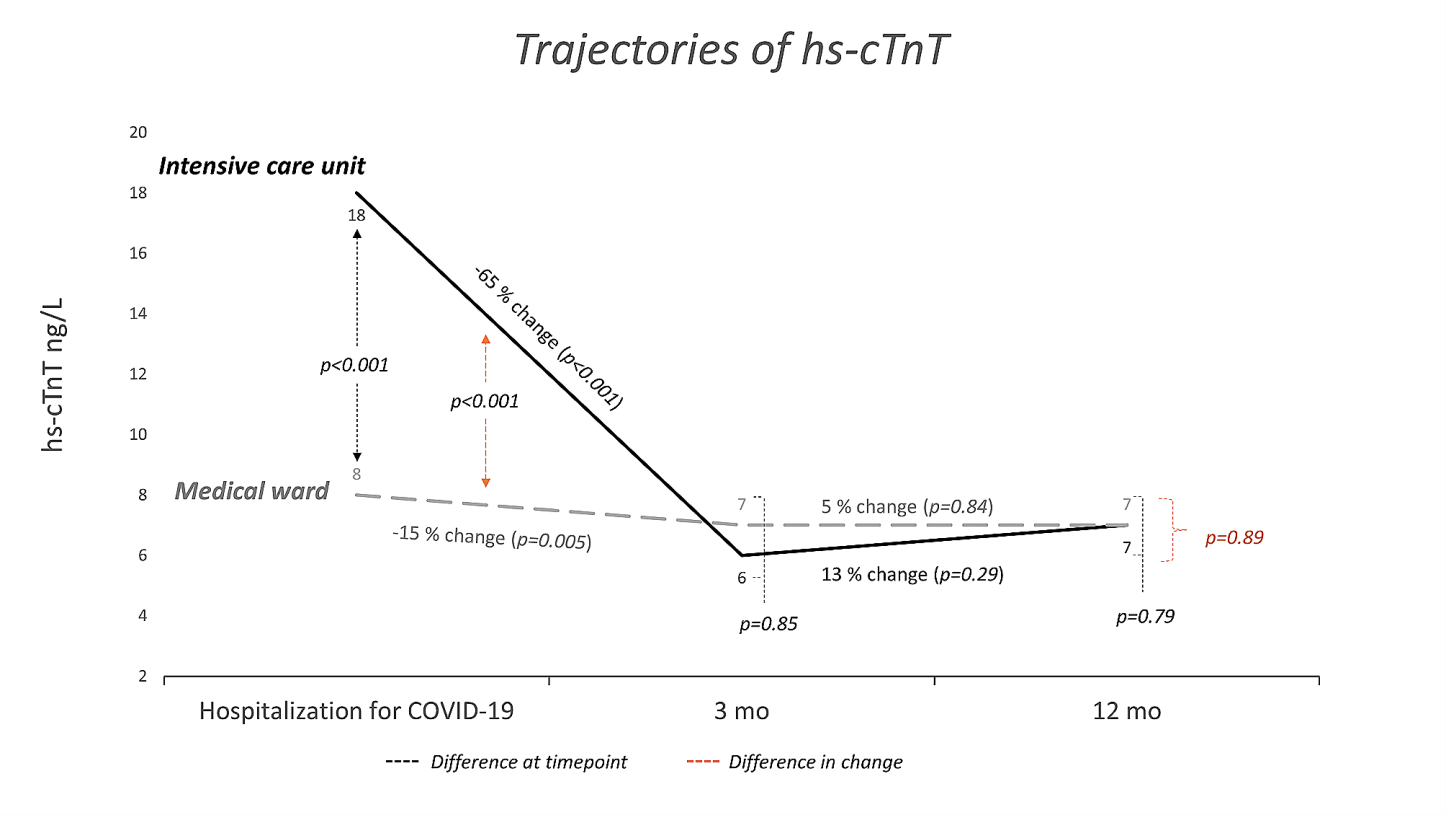
Trajectory of high sensitivity cardiac troponin T (hs-cTnT) from peak concentration during hospitalization for COVID-19 to 3 and 12 months after discharge. Stratified by admission to the intensive care unit or medical ward and presented as geometric mean. Patients with hs-cTnT available at all time points (*n* = 125). Hs-cTnT concentrations with the corresponding 95% confidence interval reported in Suppl.Table 8

Of the 189 patients enrolled, 125 (66%) had hs-cTnT measurements at all three time points (during the index hospitalization, after 3 months, and after 12 months). Patients with cardiac abnormalities had a significantly higher value of hs-cTnT at all three time points **(**Fig. 1, Suppl. Table 8A). hs-cTnT decreased significantly from hospitalization to 3 months for the overall population (geometric mean (95% CI) change − 29% (-40% to -16%), *P* < 0.001). This decrease was less pronounced and not significant in patients with cardiac abnormalities after 3 months: -18% (-40% to + 11%), *P* = 0.12). Patients treated in the ICU experienced a greater decrease in hs-cTnT to 3 months after COVID-19 compared to those treated in the medical ward: -65% (-88% to -47%) vs. -15% (-28% to + 1% %), *P* < 0.001 (Fig. 2, Suppl. Table 8B). At 3 months, the hs-cTnT concentrations were similar in patients treated at the ICU and the medical ward: geometric mean (95% CI) 6 (5–9) ng/L and 7 (6–8) ng/L, respectively (*P* = 0.99). There was no significant change in hs-cTnT from 3 to 12 months in the overall population, stratified by the presence of cardiac abnormalities or stratified for ICU treatment.

## Discussion

We report four main findings from our study: (1) hs-cTnT concentrations 3 and 12 months after COVID-19 were cross-sectionally associated with cardiac abnormalities. In contrast, peak hs-cTnT during index hospitalization was not associated with subsequent cardiac abnormalities. (2) hs-cTnT decreased by approximately 30% from hospitalization (8 ng/L) to 3 months (6 ng/L) after discharge, with greater reductions in patients receiving ICU treatment (65% decrease, from 18 ng/L to 6 ng/L)). (3) hs-cTnT < 5 ng/L at 3 months had a high NPV for ruling out cardiac pathology in the convalescent phase of COVID-19 in the total cohort as well as in the subgroup with persistent dyspnea. (4) There was no significant change in hs-cTnT from 3 to 12 months after COVID-19, irrespective of persistent dyspnea or cardiac abnormalities.

It remains unclear whether higher concentrations of troponin during acute COVID-19 are associated with structural cardiac damage in the convalescence period [26–28] or if it is mainly related to the initial disease severity [5, 29], co-morbidities [30], or a combination of these. In our study, we found that peak hs-cTnT levels during hospitalization were *not* associated with subsequent structural cardiac disease or decline in cardiac function in the convalescent phase, while it was closely associated with the severity of the infection. Moreover, hs-cTnT was not independently associated with the presence of myocardial scars on CMR, which could be explained by the limited number of patients with scars and the fact that most were minor scars. Both severe COVID-19 and non-COVID-19 infections are associated with elevated hs-cTnT [31], and as expected we found that patients treated in the ICU had higher values of hs-cTnT compared to those treated in the medical ward. In survivors of severe COVID-19, hs-cTnT levels decreased substantially in our study, and by 3 months there were no differences in hs-cTnT between patients treated in the ICU vs. the medical ward. This indicates that the severity of the infection is a significant factor contributing to the release of troponin during hospitalization and that it not necessarily reflect persistent cardiac damage. We found that patients with cardiac abnormalities had significantly higher hs-cTnT concentrations both during COVID-19 and in the convalescent phase, compared to patients without cardiac abnormalities. The decline in hs-cTnT was consistent irrespective of cardiac pathology, implying again that the cardiac injury primarily relates to the critical illness rather than direct cardiac involvement from COVID-19. Hence, the elevated hs-cTnT values could be a consequence of pre-existing undetected cardiac conditions, coupled with the systemic impact of COVID-19, which might intensify underlying issues like coronary disease, atrial fibrillation, or latent heart failure. Although inconsistent evidence on the implications of high hs-cTnT in COVID-19 [31], it does not appear unique to this disease, as non-COVID ARDS patients have similar hs-cTnT levels as COVID-ARDS [31].

We have previously reported that there is no significant change in cardiac structure and function from 3 to 12 months after hospitalization with COVID-19 [18]. In this study, we add to these findings by demonstrating stable levels of hs-cTnT from 3 to 12 months. This may imply that the cTnT levels have reached baseline within 3 months, regardless of COVID-19 severity, cardiac pathology, or persistent symptoms. This is in line with the observation that hs-cTnT at 3 months, in contrast to peak hs-cTnT while hospitalized, was associated with cardiac structure and function and that chronically elevated hs-cTnT concentrations reflect permanent cardiac abnormalities in contrast to peak hs-cTnT during hospitalization which is exaggerated by the infection itself.

Hs-cTnT ≥ 5 ng/L at 3 months was associated with cardiac pathology but was largely mediated by age and sex. Since there is an established link between hs-cTnT and cardiac remodeling [2], the non-significant association in our study is probably a result of a modest statistical power. Nonetheless, we found a high NPV of hs-cTnT < 5 ng/L for ruling out abnormal cardiac structure and function. Many patients report symptoms persisting for more than four weeks after COVID-19, a condition known as PACS. Although there were initial concerns for cardiovascular disease as a potential cause, there are few studies supporting this.In our study, 44% reported dyspnea 3 months after hospitalization, a key symptom in PACS. Despite a limited association between persistent dyspnea and cardiac pathology [18], many of these patients undergo cardiac testing. Our findings of similar hs-cTnT concentrations between patients with and without dyspnea further strengthen the hypothesis that PACS is not related to cardiac injury or dysfunction. Still, some patients have structural or functional cardiac abnormalities that may require further follow-up (whether or not it is due to COVID-19), and measurements of hs-cTnT may be useful in selecting patients in need of cardiac imaging.

### Strengths & limitations

This study was a prospective study of unselected patients hospitalized for COVID-19, and in this way minimizes selection bias, a prominent limitation in biomarker studies of COVID-19. Although only 8 patients died during the hospitalization for COVID-19, this introduces some degree of survival bias as these patients had higher hs-cTnT levels than survivors attending the 3-month visit. All patients were enrolled in the early stage of the pandemic and was unvaccinated and infected with different SARS-CoV-2 variants than the strains that are prevalent today. This may limit the external validity of our results to contemporary care. Unfortunately, since the cardiac health of the participants prior to COVID-19 was unknown for most patients, it is not possible to determine the reasons for the cardiac abnormalities that were detected. Since the patients were included early in the pandemic, a time characterized by many patients and logistical challenges, baseline echocardiography during hospitalization was not conducted as part of this study.

## Conclusion

Elevated hs-cTnT concentrations during COVID-19 hospitalization are transient, associated with the severity of the infection, and reach stable values within 3 months after discharge. Levels of hs-cTnT in the convalescent phase, in contrast to levels during hospitalization, are associated with the presence of cardiac abnormalities, and very low concentrations (< 5 ng/L) seem to reliably rule out cardiac pathology after COVID-19, regardless of persistent dyspnea. Measurements of hs-cTnT may be a useful tool in selecting patients with persistent symptoms for cardiovascular imaging after COVID-19.

### Electronic supplementary material

Below is the link to the electronic supplementary material.

Supplementary Material 1

## Data Availability

The datasets generated and/or analyzed during the current study are not publicly available due to privacy or ethical restrictions but are available from the corresponding author on reasonable request.
