# Supplementary material for: Trajectory of cardiac troponin T following moderate-to-severe COVID-19 and the association with cardiac abnormalities
Source: BMC Cardiovasc Disord. 2024 Apr 13;24:206. doi: 10.1186/s12872-024-03854-7 (PMC11015606; doi:10.1186/s12872-024-03854-7)
Supplement: Supplementary file 1 — Supplementary Material 1 [file 12872_2024_3854_MOESM1_ESM.docx]

Supplemental Material

to

**Trajectory of cardiac troponin T following moderate-to-severe COVID-19 and the association with cardiac abnormalities**

**Suppl Table 1. Predictors of higher concentrations of hs-cTnT** **3 months after COVID-19**

hs-cTnT levels were log-transformed, analyzed by multivariable linear regression and presented as effect estimates (% change per unit increase).

|  | Effect estimates (95% CI) (n=181, adjusted R^2^=0.49) | \|Z\| | P-value |
| --- | --- | --- | --- |
|  |  |  |  |
| Age (per 10 years) | 48% (38%, 59%) | 8.97 | <0.001 |
| Sex (male) | 70% (45%, 96%) | 5.45 | <0.001 |
| Caucasian  ethnicity | 8% (-25%, 41%) | 0.47 | 0.64 |
| Body mass index | -3% (-8%, 1%) | -1.40 | 0.16 |
| Obesity (BMI >30) | 32% (-15%, 79%) | 1.35 | 0.29 |
| Cardiovascular disease | 37% (-7%, 81%) | 1.64 | 0.10 |
| Hypertension | 10% (-18%, 38%) | 0.68 | 0.50 |
| Diabetes | 0% ( -18%, 38%) | 0.02 | 0.98 |
| Chronic obstructive pulmonary disease | 67% ( -6%, 140%) | 1.80 | 0.07 |
| Chronic kidney disease | -7% (-108%, 95%) | -0.14 | 0.89 |
| Current or previous smoking | 14% (-12%, 41%) | 1.07 | 0.28 |

**Suppl Table 2, Echocardiographic measurements in patients free of known cardiovascular disease**

Categorized by high sensitivity cardiac troponin T (hs-cTnT) <5 ng/L and ≥ 5 ng/L at the 3-month follow-up. Reported as mean ± SD or number (%). P values are calculated from logistic regression.

|  | cTnT <5 n=39 | cTnT ≥ 5 ng/L n=131 | P-value | Adjusted P-value  (Age + Sex) |
| --- | --- | --- | --- | --- |
| Left ventricular mass index (g/m2) | 60 ± 13 | 72 ±17 | <0.001 | 0.010 |
| Left ventricular end-diastolic volume index (ml/m^2^) | 50 ± 9 | 53 ±14 | 0.28 | 0.14 |
| Left ventricular ejection fraction (%) | 58 ± 4 | 58 ±5 | 0.57 | 0.69 |
| Left ventricular global longitudinal strain (%) | 19.9 ± 1.6 | 19.2 ±2.2 | 0.14 | 0.16 |
| Left atrial volume index (ml/m2) | 24 ± 7 | 27 ±9 | 0.07 | 0.50 |
| E / e` | 7.2±2.7 | 8.3 ±2. | 0.05 | 0.57 |
| Right ventricular basal diameter (cm) | 3.5 ± 0.6 | 3.7 ±0.5 | 0.04 | 0.26 |
| Tricuspid annular plane systolic excursion (cm) | 2.4 ± 0.3 | 2.4 ±0.3 | 0.57 | 0.88 |
| Right ventricular free wall strain (%) | 26.4 ± 2.8 | 25.9 ±4.2 | 0.49 | 0.39 |
| Estimated systolic pulmonary arterial pressure (mmHg) | 25 ± 6 | 23 ±9 | 0.22 | 0.026 |
| Late gadolinium enhancement (n=58) | 1 (6%) | 11(27%) | 0.08 | 0.41 |

**Suppl. Table 3. Changes in echocardiographic measurements from 3 to 12 months after COVID-19**

Stratified by high sensitivity cardiac troponin T (hs-cTnT) levels of 5 ng/L at 3 months.

|  | hs-cTnT <5 ng/L n=39 | hs-cTnT ≥ 5 ng/L  n=150 | P-value | Adjusted P-value  (Age + Sex + CVD) |
| --- | --- | --- | --- | --- |
| Δ Left ventricular mass index (g/m2) | -0.3 ± 6.9 | -1.7 ±9.5 | 0.41 | 0.35 |
| Δ Left ventricular end-diastolic volume index (ml/m^2^) | -1.5 ± 4.8 | 0.4 ±7.9 | 0.19 | 0.23 |
| Δ Left ventricular ejection fraction (%) | 0.6 ± 2.6 | -0.0 ±3.5 | 0.29 | 0.91 |
| Δ Left ventricular global longitudinal strain (%) | 0.1 ± 1.9 | 0.1 ±1.8 | 0.90 | 0.71 |
| Δ Left atrial volume index (ml/m2) | -0.2 ± 4.2 | 0.1 ±5.6 | 0.79 | 0.74 |
| Δ E / e` | 0.1 ± 2.6 | -0.4 ±2.5 | 0.32 | 0.55 |
| Δ Right ventricular basal diameter (cm) | -1.0 ± 0.3 | -1.0 ±0.3 | 0.56 | 0.77 |
| Δ Tricuspid annular plane systolic excursion (cm) | -0.0 ± 0.2 | -0.0 ±0.2 | 0.78 | 0.62 |
| Δ Right ventricular free wall strain (%) | 0.0 ± 2.3 | 0.1 ±3.1 | 0.87 | 0.59 |
| Δ Estimated systolic pulmonary arterial pressure (mmHg) | -3.8 ± 6.0 | -0.7 ±10.5 | 0.20 | 0.54 |

**Suppl. Table 4. Changes in echocardiographic measurements from 3 to 12 months after COVID-19 in patients without pre-existing cardiovascular disease.**

Stratified by high sensitivity cardiac troponin T (hs-cTnT) levels of 5 ng/L at 3 months.

|  | hs-cTnT <5 ng/L n=39 | hs-cTnT ≥ 5 ng/L  n=150 | P-value | Adjusted P-value  (Age + Sex) |
| --- | --- | --- | --- | --- |
| Δ Left ventricular mass index (g/m2) | -0.3 ± 6.9 | -2.1 ±10.9 | 0.26 | 0.43 |
| Δ Left ventricular end-diastolic volume index (ml/m^2^) | -1.4 ± 4.6 | 0.4 ±7.6 | 0.22 | 0.30 |
| Δ Left ventricular ejection fraction (%) | 0.7 ± 2.7 | 0.3 ±3.7 | 0.58 | 0.99 |
| Δ Left ventricular global longitudinal strain (%) | 0.2 ± 2.0 | 0.2 ±1.6 | 0.94 | 0.98 |
| Δ Left atrial volume index (ml/m2) | 0.2 ± 4,0 | -0.1 ±5.5 | 0.76 | 0.58 |
| Δ E / e` | 0.3± 2.3 | -0.1 ±2.0 | 0.48 | 0.91 |
| Δ Right ventricular basal diameter (cm) | -0.0 ± +0.2 | -0.0 ±0.4 | 0.56 | 0.40 |
| Δ Tricuspid annular plane systolic excursion (cm) | -0.0 ± 0.2 | -0.0 ±0.2 | 0.67 | 0.38 |
| Δ Right ventricular free wall strain (%) | -0.0 ± 2.3 | -0.0 ±2.9 | 0.98 | 0.93 |
| Δ Estimated systolic pulmonary arterial pressure (mmHg) | -4.0 ± 6.3 | -2.1 ±9.8 | 0.47 | 0.57 |

**Suppl. Table 5. Performance of hs-cTnT in detecting cardiac abnormalities**

Sensitivity, specificity, negative predictive value, and positive predictive value for cardiac abnormalities using a high sensitivity cardiac troponin T threshold of 5 ng/L in the total study population and in patients without pre-existing cardiovascular disease.

|  | Total population, n=189 | Without CVD, n=170 |
| --- | --- | --- |
| Sensitivity | 94% (90-97% | 92% (88-96%) |
| Specificity | 26% (19-32%) | 27% (20-34%) |
| Positive predictive value | 30% (24-37%) | 26% (19-33%) |
| Negative predictive value | 92% (89-96%) | 92% (88-96%) |

**Suppl. Table 6. Cardiac abnormalities after COVID-19 in patients without pre-existing cardiovascular disease**
Assessed by echocardiography and cardiac magnetic resonance in patients with high sensitivity cardiac troponin T (hs-cTnT) <5 ng/L and ≥ 5 ng/L. Comparisons between the groups are performed with logistic regression.

| *Echocardiography (n=189)* | hs-cTnT<5  n=39 | hs-cTnT≥5  n=131 |
| --- | --- | --- |
| Left ventricular hypertrophy | 0 (0%) | 4 (3 %) |
| Diastolic dysfunction | 2 (6%) | 17 (14%) |
| Systolic dysfunction | 0 (0%) | 6 (5 %) |
| Right ventricular dysfunction | 0 (0%) | 5 (5%) |
| Abnormal cardiac findings with echocardiography | **2 (5%)** | **29 (22%)** |
| Unadjusted | OR 5.3 (95% CI 1.3-23.1) P=0.031 | |
| Adjusted for age and sex | OR 2.4 (95% CI 0.5-11.9)  P=0.27 | |
| *Cardiac magnetic resonance (n=58)* | **n=17** | **n=41** |
| Myocardial scar (LGE) | 1 (6%) | 10 (26%) |
| Abnormal cardiac findings with echocardiography and cardiac magnetic resonance | **3 (8%)** | **45 (26%)** |
| Unadjusted | OR 4.2 (95% CI 1.2-14.5)  P=0.023 | |
| Adjusted for age and sex | OR 1,9 (95% CI 0.5-7.2)  P=0.35 | |

**Suppl. Table 7. Performance of hs-cTnT in detecting cardiac abnormalities in patients with dyspnea**

Sensitivity, specificity, negative predictive value, and positive predictive value for abnormal cardiac findings using a high sensitivity cardiac troponin T (hs-cTnT) threshold of 5 ng/L in patients with dyspnea (modified Medical Research Council (mMRC) ≥1 and mMRC ≥2 in the total study population and in patients without pre-existing cardiovascular disease.

|  | Total population, n=189 | | Without CVD, n=170 | |
| --- | --- | --- | --- | --- |
|  | Dyspnea, mMRC≥1 | Dyspnea, mMRC≥2 | Dyspnea, mMRC≥1 | Dyspnea, mMRC≥2 |
| Sensitivity | 95% (91 - 99%) | 93% (86 - 99%) | 95% (91 - 99%) | 93% (86 - 99%) |
| Specificity | 25% (17 -33%) | 11% (3-18%) | 25% (17 -33%) | 11% (3-18%) |
| Positive predictive value | 24% (16 - 32%) | 24% (13 - 35%) | 24% (16 - 32%) | 24% (13 - 35%) |
| Negative predictive value | 96% (93 - 99%) | 83% (74 - 93%) | 96% (93 - 99%) | 83% (74 - 93%) |

**Suppl. Table 8: Hs-cTnT concentrations during hospitalization for COVID-19, after 3 months and after 12 months, and the relative changes between these time points.**

Patients are categorized based on hospital treatment (ICU vs medical ward; Panel A) and the presence of cardiac abnormality (Panel B). Values are presented as geometric mean (95% CI) and ratios as % change (95% CI). P values are calculated by the Mann-Whitney U test for paired samples and Wilcoxon signed-rank test for the significance of changes

**A**

|  | Cardiac abnormality  n=34 | No cardiac abnormality  n=91 | P-value |
| --- | --- | --- | --- |
| Hospitalization peak hs-cTnT (ng/L) | 14 (10-21) | 8 (7-10) | <0.001 |
| 3-month hs-cTnT (ng/L) | 12 (9-15) | 6 (5-7) | <0.001 |
| 12-month hs-cTnT (ng/L) | 12 (9-15) | 6 (5-7) | <0.001 |
| Relative change from hospitalization to 3 months | -18% (-40,11) | -32% (-45,-17) | 0.14 |
| Relative change from hospitalization to 3 months | 1% (-13,18) | 8% (-4,22) | 0.68 |

**B**

|  | ICU  n=25 | Medical ward  n=100 | P-value |
| --- | --- | --- | --- |
| Hospitalization peak hs-cTnT (ng/L) | 18 (11-29) | 8 (7-10) | <0.001 |
| 3-month hs-cTnT (ng/L) | 6 (5-9) | 7 (6-8) | 0.85 |
| 12-month hs-cTnT (ng/L) | 7 (6-9) | 7 (6-8) | 0.79 |
| Relative change from hospitalization to 3 months | -65% (-78, -47) | -15% (-28,1) | <0.001 |
| Relative change from hospitalization to 3 months | 13% (-15,49) | 4% (-5,16) | 0.89 |

**Suppl. Figure 1** Flowchart participants with echocardiography and hs-cTnT.

**
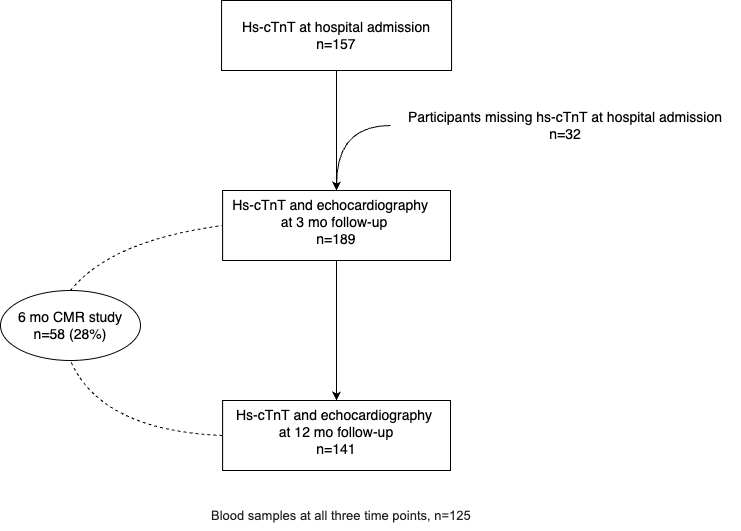
**
